# Transcriptomic response of yeast cells to *ATX1* deletion under different copper levels

**DOI:** 10.1186/s12864-016-2771-6

**Published:** 2016-07-11

**Authors:** Ayca Cankorur-Cetinkaya, Serpil Eraslan, Betul Kirdar

**Affiliations:** Department of Chemical Engineering, Faculty of Engineering, Bogazici University, 34342 Istanbul, Turkey; Present address: Cambridge Systems Biology Centre & Department of Biochemistry, University of Cambridge, CB2 1GA Cambridge, United Kingdom; Present address: Diagnostic Centre for Genetic Diseases, Koc University Hospital, Davutpasa Cd. No:43010 Topkapı, Istanbul, Turkey

**Keywords:** Copper transport, Copper homeostasis, Cell cycle regulation, Wilson disease, Menkes disease, Yeast, Copper chaperon

## Abstract

**Background:**

Iron and copper homeostatic pathways are tightly linked since copper is required as a cofactor for high affinity iron transport. Atx1p plays an important role in the intracellular copper transport as a copper chaperone transferring copper from the transporters to Ccc2p for its subsequent insertion into Fet3p, which is required for high affinity iron transport.

**Results:**

In this study, genome-wide transcriptional landscape of *ATX1* deletants grown in media either lacking copper or having excess copper was investigated. *ATX1* deletants were allowed to recover full respiratory capacity in the presence of excess copper in growth environment. The present study revealed that iron ion homeostasis was not significantly affected by the absence of *ATX1* either at the transcriptional or metabolic levels, suggesting other possible roles for Atx1p in addition to its function as a chaperone in copper-dependent iron absorption. The analysis of the transcriptomic response of *atx1∆/atx1∆* and its integration with the genetic interaction network highlighted for the first time, the possible role of *ATX1* in cell cycle regulation, likewise its mammalian counterpart *ATOX1*, which was reported to play an important role in the copper-stimulated proliferation of non-small lung cancer cells.

**Conclusions:**

The present finding revealed the dispensability of Atx1p for the transfer of copper ions to Ccc2p and highlighted its possible role in the cell cycle regulation. The results also showed the potential of *Saccharomyces cerevisiae* as a model organism in studying the capacity of *ATOX1* as a therapeutic target for lung cancer therapy.

**Electronic supplementary material:**

The online version of this article (doi:10.1186/s12864-016-2771-6) contains supplementary material, which is available to authorized users.

## Background

Copper plays an important role in the oxidation and reduction reactions with its ability to accept and donate electrons. This ability also makes the maintenance of the homeostasis of this metal vital since it can be toxic to cells when present in excess, by inducing the formation of toxic reactive oxygen species. Thus, organisms contain various metal homeostasis factors to control the cellular accumulation, distribution, and sequestration of the metal, which is evolutionarily conserved [1]. This makes *Saccharomyces cerevisiae* an ideal model organism to investigate the copper metabolism in relation with the iron and oxygen radical metabolisms which show high overlap.

Copper is required for the oxidase activity of Fet3p [2]. The requirement of copper for trafficking of Fet3p/Ftr1p complex to the plasma membrane under iron deprived conditions represents the importance of copper for the maintenance of iron homeostasis in yeast [3]. The copper delivery pathway that mediates the transport of copper to Fet3p includes Ctr1p, Atx1p and Ccc2p [4]. Ctr1p is the high affinity copper transporter, which transfers the reduced copper by membrane reductases; Fre1p and Fre2p. The expression of *CTR1* is regulated by copper level through Mac1p. It is induced under low copper conditions and repressed by high copper levels to reduce copper uptake [3]. Ctr1p is also involved in the transport of copper ions to secretory pathway. Copper is transported to cytosolic copper chaperone Atx1p, which delivers copper to Ccc2p. Atx1p was first identified as multi-copy suppressor of oxygen toxicity in superoxide dismutase (SOD) deficient cells [5]. However, this observation was shown to be not valid when the cells were treated with copper chelator. The decreased resistance to oxygen under copper deprived conditions were reversed by addition of copper. Copper dependent bypass of SOD deficiency and also the presence of copper ion binding domains in Atx1p indicated that this protein is involved in the intracellular transport and sequestration of copper [5].

*ATX1* deleted cell exhibited no growth on iron deficient medium similar to the *CTR1* and *CCC2* deletion mutant, which are defective in high affinity iron uptake. Measurement of ferrous iron uptake of *ATX1* deleted cells also showed a reduction, which could be restored by copper supplementation. These results indicated that Atx1p operates in the same way as Ctr1p and Ccc2p to deliver copper to Fet3p. Studies also showed that overexpression of *CCC2* can suppress the effect of absence of *ATX1* gene but the overexpression of *ATX1* gene did not rescue the iron deficiency in *CCC2* deleted strains. This finding revealed that Ccc2p functions downstream of Atx1p and there is an Atx1p-independent pathway that can reverse the effect of absence of *ATX1* when *CCC2* is overexpressed. Observation of a more severe growth defect in the temperature sensitive double deletion mutant of *ATX1* and *END3*, absence of which blocks endocytosis, on iron deficient medium indicated that endocytosis might be the Atx1p-independent pathway that delivers copper to intracellular locations [4].

*ATOX1* is the human ortholog of *ATX1* and has a similar function in human. Atox1p directly interacts with the proteins encoded by *ATP7A* and *ATP7B*, which are the Menkes and Wilson disease causing genes and delivers copper to these proteins [6]. Studies performed with Atox1 deficient mice cells demonstrated that absence of *ATOX1* leads to copper accumulation due to impaired copper efflux [7]. Although no disease mutations have been reported in *ATOX1* [8], Menkes disease like phenotype was observed in *ATOX1* knockout mice [7].

This study concerns with the transcriptional response of the yeast cells to the absence *ATX1* gene, which is the yeast ortholog of *ATOX1* gene in human, under different amounts of copper containing conditions. Atx1p and Ccc2p are reported to be two consecutive proteins in the intracellular transport of copper. The presence of a parallel copper trafficking pathway through endocytosis was proposed by genetic analysis but the exact mechanism still remains elusive (4). The aim of the present study was to investigate the genome-wide effects of the absence of *ATX1* gene under conditions lacking copper and containing high levels of copper. The analyses of the significantly expressed genes in response to the deletion of *ATX1* gene under changing copper conditions in comparison to the reference strain showed that the absence of *ATX1* gene resulted in a different transcriptional re-organization than that of *CCC2* deletion supporting the presence of an Atx1p independent pathway that delivers copper to Ccc2p. Analysis of the transcriptome data and also further integrative analyses with genetic interaction and regulatory network highlighted a potential role of *ATX1* gene in the cell cycle regulation as its mammalian counterparts.

## Methods

### Strains and growth media

Homozygous *atx1*Δ*/atx1*Δ strains of *S. cerevisiae* from a genetic background of BY4743 (*MAT*a*/MAT*Δ *his3*Δ*1*/*his3*Δ*1 leu2*Δ*0*/l*eu2*Δ*0 lys2*Δ*0*/+ *met15*Δ*0*/+ *ura3*Δ*0*/*ura3*Δ*0*) were purchased from the European *Saccharomyces cerevisiae* Archive for Functional Analysis. Synthetic defined medium [9] without CuSO_4_ (copper deficient condition) and with 0.5 mM CuSO_4_ supplementation (high copper level) was used in the fermentations.

### Batch culture, sample description and microarray hybridization

The fermentation experiments were carried out in duplicates using B-Braun Biostat B plus fermenters with 1.5 L working volume at two different levels of synthetic defined medium at 30 °C and an agitation rate of 800 rpm. The pH of the culture was controlled at 5.5 with 1 M NaOH and HCl. The fermentations were conducted under fully aerated conditions with an aeration rate of 1 vvm. RNA samples were collected at mid-exponential phase at an OD range of 0.7–0.8. The samples were immediately frozen in liquid nitrogen and stored at −80 °C until further processing. The enzymatic lysis protocol of RNeasy mini kit (Qiagen, USA) with robotic workstation QIAcube (Qiagen, USA) was used for the isolation of RNA from the samples. RNA integrity was determined by Bioanalyzer 2100 (Agilent Technologies, USA) using RNA6000 Nanokit (Agilent Technologies, USA) as described by the manufacturer. The RIN values of the samples were between 7 and 10. NanoDrop (ND-1000, Thermo Scientific) was used for the quantitative and qualitative analyses of RNA.

Microarray hybridization was performed as described previously [10]. Briefly, 100 ng of total RNA was used to synthesize first-strand cDNA then to convert into a double-stranded DNA using GeneChip® 3’ IVT Express Kit (Affymetrix Inc., U.S.A). This double stranded cDNA was used as a template for in vitro transcription and synthesis of biotin-labelled aRNA. The final product was purified and quantified using the Nanodrop spectrophotometer before fragmentation. GeneChip reagents were used for the purification and 15 fragmentation steps. Agilent 2100 Bioanalyzer was used for the fragmented aRNA evaluation. (Agilent Technologies, Germany). The reagents supplied in the GeneChip® Hybridization, Wash, and Stain Kit were used to prepare the Affymetrix Yeast 2.0 arrays for hybridization. A total of 5 μg of aRNA was loaded onto 169 format arrays and hybridized for 16 h. The chips were then loaded into a fluidics station for washing and staining using Affymetrix Command Console® Software (AGCC) 3.0.1 Fluidics Control Module with Mini_euk2v3. Finally, the chips were loaded onto the Affymetrix GeneChip Scanner 3000. All applications were performed as described in the Affymetrix GeneChip®Expression Analysis Technical Manual.

### Intracellular iron determination and sample preparation

Samples (24 ml) were collected from the cell cultures that were grown until mid-exponential phase in the Erlenmeyer flasks under copper deficient and high copper conditions. The samples were centrifuged at 400 g for 3 min at 4 °C. The cell pellets were washed with 1 mM KCN and centrifuges at 400 g for 3 min. After digestion in 5 ml of nitric acid at 100 °C for 2 h, the samples were centrifuged at 10000 g for 2 min and diluted with 5 ml of water. The intracellular and extracellular iron concentrations were kindly determined by Redokslab Analytical Systems Inc., Istanbul, Turkey, using iCap Q ICP-MS (Thermo Scientific Inc., USA).

### Microarray data acquisition and analysis

The cell files generated by Microarray Suite v5.0 were pre-processed with dChip software [11] using the perfect match (PM)-miss match (MS) difference model to obtain the expression levels of transcripts. These values were normalized by a baseline array with a median overall intensity. The array corresponding to reference strain with high copper condition was selected as the baseline array. The presence of single, array and probe outliers was checked and no outlier chips were identified.

The statistical analyses of the microarray data were performed using MATLAB. Significantly and differentially expressed genes in response to the deletion of *ATX1* gene under changing copper levels in comparison to reference strain, were identified by two-way Analyses of Variance (2-way-ANOVA) and fold change analyses, respectively. The threshold to determine the differentially expressed genes were set to 1.5 whereas significance threshold was taken as 0.05. The significantly and differentially expressed genes were analysed in four distinct groups. The first group, G1, included the genes that were significantly expressed only in response to the deletion of *ATX1* gene and that also showed a fold change above the threshold between the reference strain and deletion mutant either under conditions lacking copper or containing high levels of copper. The second group, G2, contained the genes, which were significantly expressed only in response to copper level and which were differentially expressed between copper deficient and high levels of copper containing conditions either in the reference strain or in the deletion mutant. The third group, G3, included the genes, which were significantly and differentially expressed both in response to gene deletion and change in the copper level in an additive manner. The last group, G4 were composed of the genes, which were affected from the interactive effect of gene deletion and copper level and which showed differential expression either in response to copper or gene deletion. The significantly enriched GO biological process ontology terms (*p*-value < 0.05) in each group were determined using GO Term Finder [12]. The microarray data for the *ATX1* deleted cells has been submitted to ArrayExpress at the European Bioinformatics Institute under accession number [E-MEXP-4230] in compliance with MIAME guidelines. The data for the reference strain can be found in the ArrayExpress at the European Bioinformatics Institute under accession number [E-MEXP-3927].

### RT-qPCR study

The expression level of *CLN3*, *IME1*, *MSA1*, *MSA2*, *PCL9*, *SIC1*, and *ACE2* were determined under copper deficient and high levels of copper containing conditions in the reference strain and in the *ATX1* deleted cells.

The primers were designed using Primer3 software, 18–24 bases in length, with a GC content between 50 to 60 % and T_m_ in the range of 55–58 °C. The complete set of primer sequences were provided in Additional file 1.

The amplicon size was determined to be either between 100 and 150 or 200 and 250 base pairs long. In order to unify the initial concentration of RNA in the analyses, all samples were diluted to the same concentration (35 ng/μl) prior to the real-time RT-qPCR application. Reverse transcription was carried out at 50 °C for 30 min. QuantiTect RT mix (Qiagen, USA, Cat no: 204245) was used at a ratio of 0.01 total reaction volume, which was 12.5 μl. Assays were conducted in duplicates. The synthesized cDNA template was immediately allowed to proceed with the polymerase chain reaction. Qiagen QuantiTect® SYBR® Green one step RT-qPCR kit was used for real-time RT-qPCR applications as described by the manufacturer (Qiagen, USA, Cat no: 204245). All kit contents are optimized and validated by the manufacturer. The PCR reactions were performed in a final reaction volume of 12.5 μl containing the final concentration of 2.5 mM of MgCl_2_ and 0.5 mM of forward and reverse primers. Plates and adhesive seals were manufactured by Bio-Rad Laboratories (Cat no: MSB1001, MLP9601). The reaction mixtures were prepared manually and the reactions were allowed to proceed in iCycler 5 instrument (Bio-Rad Laboratories).

iCycler™ iQ Optical System Software version 3.0a (Bio-Rad Laboratories) was used with PCR base line subtracted curve fit method for the measurement of quantification cycle (Cq). Raw Cq values were provided in Additional file 2. The housekeeping genes that were used to normilisation were selected using the microarray data. *ARF1*, *TDH3* and *FBA1* were identifed to be the most stable three genes among the candidate houseekeeping gene set [13] and the geometric average of the Cq values of these genes were used for normalisation. 2^−ΔΔC^_T_ method was used to determine the fold change between the conditions for genes of interest [14].

### Integration of transcriptome data with genetic interaction and regulatory network

The genetic interactions reported in the BioGrid database (Release 3.3.123) [15] were used for the construction of genetic interaction network. The network for the genes, which are responsive to the deletion of *ATX1*, was constructed using the genetic interaction between the genes that take place either in G1, G3 or G4. The complete genetic interaction network is constructed only for the genes that exist in the transcriptome data. Cytoscape [16] was used for the visualization and removal of the duplicate edges within the constructed network.

The reported TF-protein interactions with a documented evidence in Yeastract database [17] were used for the construction of regulatory network. The transcription factors that regulate the genes in group G1, G3 and G4 were identified from the network. The number of the genes that each TF regulates in the whole network as well as in the group of G1, G3 and G4 were determined. Enrichment analysis was conducted to identify the over-represented TFs for the genes that are responsive to the deletion of *ATX1* using hypergeometric distribution function. The TFs with Bonferroni corrected *p*-value < 0.0001 were defined as significantly enriched TFs.

## Results

Cytosolic copper metallochaperon; Atx1p, is known to transport copper to Ccc2p for eventual insertion into Fet3p and its deletion causes deficiency in iron absorption, which can be supressed by addition of copper. There is also previous evidence on the existence of an Atx1p independent mechanism that transports copper to Ccc2p [4] but the exact mechanism is still elusive. Previously, we have investigated the transcriptional reorganization of the *CCC2* deleted cells in response to different levels of copper and identified the pathways that were affected from the disturbance of the copper homeostasis due to the absence of *CCC2* gene [10]. In order to provide further insight into the role of *ATX1* and observe the genome-wide effect of its absence, the transcriptional re-organisation in response to the deletion of *ATX1* was investigated under two different copper levels. *ATX1* deletion mutant was cultivated in fully controlled fermenters in duplicates in defined medium lacking copper or containing high levels of copper (0.5 mM), which was shown to restore the decreased respiratory capacity of the *ATX1* deleted strain as it was the case for *CCC2* deleted cells [10] (Additional file 3). RNA samples were collected at the mid-exponential phase.

### Identification of significantly and differentially expressed genes

**Fig. 1:**
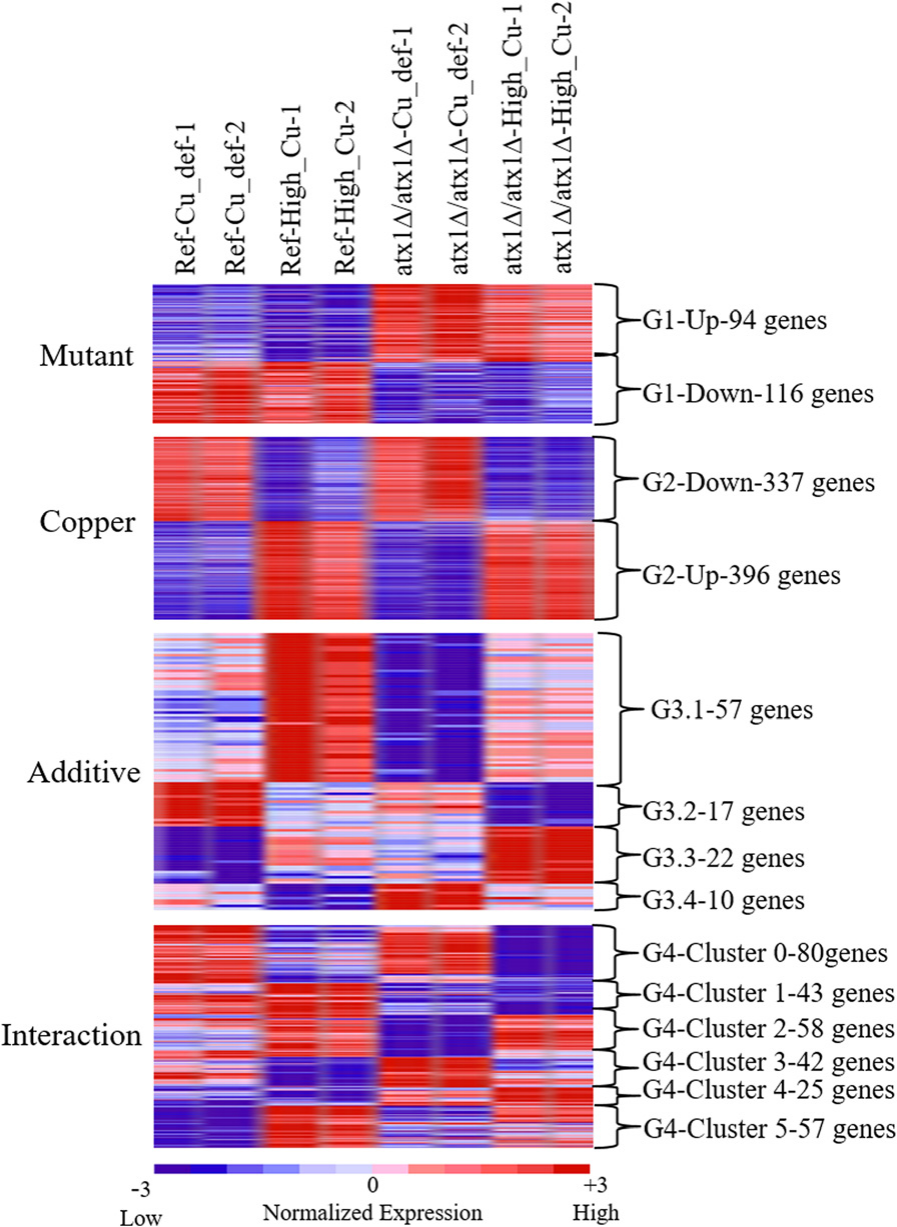
Significantly and differentially expressed genes identified within each group. The figure contains the heat map representation of the genes, which were significantly and differentially expressed in response gene deletion, changing copper levels, or their additive effect, or their interactive effect. Colour key from blue to red indicates the low to high level of expression, respectively. These genes were further grouped according to the change in the direction of expression level. The number of the genes, which were identified to be up- or down-regulated in response to gene deletion effect or high copper levels were shown next to each group. Similarly, the number of the genes within each subgroup of G3, which compose of the genes significantly and differently expressed both in response to the gene deletion and copper level and the number of the genes within each cluster of G4, which compose of the genes significantly and differently expressed both in response to the interaction effect of gene deletion and copper level were represented next to each subgroup and cluster

Genome-wide transcript levels of the *ATX1* deleted and the reference strain obtained under conditions containing two different levels of copper were analysed by 2-way ANOVA to identify the significantly expressed genes and by fold change analysis to identify the differentially expressed genes (Additional file 4). The genes, which show both significant and differential change, were classified into four groups (G1-G4) and further investigated (Fig. 1). This analysis revealed that 210 genes (116 down- and 94 up-regulated) showed both significant and differential expression only in response to deletion of *ATX1* gene when compared to the reference strain under both copper deficient and high levels of copper containing conditions (G1). A total of 733 genes (337 down- and 396 up-regulated) were differentially and significantly transcribed, in response to high copper levels (G2). G3 consisted of 106 genes, which showed differential and significant change both in response to gene deletion and change in the copper level without a significant interaction effect. This group of genes could further be classified into four different sub-groups; 57 genes (G3.1), which were down-regulated in response to the deletion of *ATX1* when compared to the reference strain under both copper levels and up-regulated in response to high copper levels in comparison to the copper deficient conditions in both strains, 17 genes (G3.2), which were down-regulated both in the *ATX1* deleted cells in comparison to the reference strain under both copper levels and under high copper containing conditions in comparison to the copper deficient conditions in both strains, 22 genes (G3.3), which were up-regulated both in the *ATX1* deleted cells in comparison to the reference strain under both copper levels and under high copper containing conditions in comparison to the copper deficient condition in both strains, and 10 genes (G3.4), which showed higher expression in the *ATX1* deleted cells compared to the reference strain under both copper levels and showed lower expression under high copper containing conditions when compared to the copper deficient conditions in both strains. The last group of genes (G4) consisted of 305 genes displaying a significant interaction effect and differential expression either in response to changing copper levels in any strain or in response to deletion of *ATX1* under any condition. The expression profiles of these genes were used to cluster these genes into 6 different clusters using Self Organizing Maps (SOM) [18] and each cluster was separately investigated. Cluster 0 composed of the genes, which showed lower level of expression under conditions containing high copper levels compared to copper deficient conditions and this response was more pronounced in the *ATX1* deleted cells. Cluster 1 contained the genes, which showed lower levels of expression under conditions containing high levels of copper when compared to copper deficient conditions in the *ATX1* deleted cells but higher levels of expression in the reference strain. Cluster 2 composed of the genes, which were down-regulated in the absence of *ATX1* gene under copper deficient conditions in comparison to the reference strain, but not responsive under high copper containing conditions. The genes, which showed the lower level of expression in the reference strain under high levels of copper containing conditions and higher level of expression in the *ATX1* deleted strain under copper deficient conditions were clustered in Cluster 3. The genes in Cluster 4 were the ones that showed higher expression under high levels of copper containing condition when compared to the copper deficient conditions in the absence of *ATX1* deleted cells but not in the reference strain. Lastly, the genes that were up-regulated under copper deficient conditions but down-regulated under high levels of copper containing conditions in the *ATX1* deleted cells when compared to the reference strain were clustered in Cluster 5.

### Effect of *ATX1* gene deletion

The genes, whose expression levels were significantly (*p*-value < 0.05) and differentially (FC > 1.5) changed only in response to the *ATX1* deletion were identified as the transcripts that are responsive to gene deletion only (G1). This analysis revealed 116 transcripts that were significantly and differentially down-regulated in the *ATX1* deleted cells when compared to the reference strain irrespective of the copper level without a significant interaction effect. These genes were identified to be significantly enriched with transcription from RNA polymerase II promoter GO biological process term (*p*-value < 0.01).

The manual investigation of the genes revealed down-regulation of the genes involved in the cell cycle regulation in response to *ATX1* deletion. The genes encoding Ime1p, which is the master regulator of meiosis, Cln3p, which is involved in the regulation of G1 to S transition, Cdc39p, which is involved in cell cycle regulation, Msa1p and Msa2p, which are activators of G1 specific transcription factors involved in the regulation of G1 to S transition in mitotic cycle, Pog1p reported to have a putative role in the cell cycle regulation were significantly and differentially down-regulated in response to *ATX1* deletion independent of the copper level. The expression levels of *CLN3*, *IME1*, *MSA1* and *MSA2* were further studied by real-time RT-qPCR (Additional file 5). These analyses confirmed the differential down-regulation of *CLN3* under high copper level containing conditions, *MSA1* under copper deficient condition and *IME1* and *MSA2* under both conditions in the absence of *ATX1* in comparison to the reference strain. Similarly, *MCM1* encoding a cell type specific transcription factor involved in the regulation of M/G1 and G2/M cell cycle and in the arginine catabolic process was observed to be significantly repressed in response to *ATX1* deletion. There were also several genes involved in the cell cycle (*VHS2, SPS1, SMC1, GIC2, PAN1, FUS3, MYO5, EGT2, CRC4, IME1, HOP2, WHI3, HRR25, SML1, SSO2, BNI5*) as well as several genes encoding cell wall proteins that were found to be repressed.

A detailed manual inspection of the down-regulated genes also revealed another important group of genes (*NAB3, NOP1, PIT1, NRD1, HRP1*) that are involved in RNA 3’ end processing. *RPO21* and *MED7* encoding the largest subunit and a subunit of mediator complex of RNA polymerase II, respectively were also down-regulated. Targets of three down-regulated genes (*SFL1, RPH1, ROX1*) were found to be significantly enriched with iron ion homeostasis and iron coordination entity transport biological process terms. *ACA1* encoding a TF involved in carbon source utilization, *ASK1* encoding a regulator of glycerol channel, which is involved in response to oxidative stress and glycerol transport, *XIR1* encoding a transcriptional repressor that regulates hypoxic genes and *MIG1* encoding a TF involved in glucose repression were also observed to have significantly reduced expression levels in the absence of Atx1p. Several genes encoding proteins involved in endocytosis and ER to Golgi transport were identified among this group of genes, which are down-regulated in response to the deletion of *ATX*1. The genes encoding a zinc transporter, Zrt3p and a zinc metalloprotease, Ste24p, a cation transporter Pmp2p and manganese ion transporter, Mam3p were also found to be negatively affected from the deletion of *ATX1*. The observation of the high number down-regulated genes encoding transcription factor or regulatory proteins within this group may possibly be related to a trans-regulatory function of Atx1p.

The 94 transcripts, which were up-regulated in *ATX1* deleted cells, were not found to be significantly enriched with any GO biological process term. Manual inspection of this group revealed that the genes encoding proteins involved in mitochondrial fatty acid synthesis (Oar1p), inner-carnitine transporter required for carnitine dependent transport of acetyl CoA from peroxisomes to mitochondria during fatty acid beta-oxidation (Crc1p), mitochondrial inter-membrane protein (Hem15p) in heme biosynthesis, mitochondrial carrier protein (Mtm1p) involved in iron homeostasis were observed to be up-regulated in response to *ATX1* deletion. Mtm1p is also manganese ion and pyridoxal phosphate transporter and has a role in the activation of mitochondrial Sod2p by facilitating the incorporation essential manganese cofactor. Another mitochondrial protein (Pet122p), which is a translational activator of Cox3p was also up-regulated. The genes associated with meiosis (*SPO75*, *MSC6*, *MER1*, *REC114* and *CSM4*), the genes involved in biotin (*BIO3*) and thiamine (*THI22*) biosynthesis were among the induced genes in response to deletion of *ATX1* gene irrespective of the copper levels.

### Effect of copper level

The genes displaying a significant (*p*-value < 0.05) and differential (FC > 1.5) expression only in response to changing copper levels were identified as copper responsive genes only (G2). 337 genes were down- and 396 genes were up-regulated under high levels of copper containing conditions in comparison to the copper deficient conditions. Down-regulated genes were significantly (*p*-value < 10^−7^) enriched with ribosome biogenesis, methylation, ncRNA processing, nitrogen compound metabolism, macromolecule methylation GO biological process terms. Up-regulated genes were found to be significantly (*p*-value < 0.05) enriched with response to stimulus and iron ion homeostasis GO biological process terms. Up-regulation of the genes encoding the high affinity iron transporter; Fet3p and siderophore iron transporters; Fth1p, Arn1p and Enb1p under high levels of copper containing conditions might indicate that high copper led to iron deficiency both in the reference and the *ATX1* deleted strain. However, analyses of the intracellular and extracellular iron levels revealed that high copper levels did not result in any significant change in the intracellular and extracellular iron levels (Additional file 6). The up-regulation of iron transporters under high levels of copper containing conditions might be as a consequence of enhanced requirement for iron as a cofactor to decompose H_2_O_2_ in response to increased oxidative stress by high levels of copper [19].

### Additive effect of changing copper levels and deletion of *ATX1* gene

Inspection of 2-way ANOVA results revealed that there were also a group of genes, whose expression showed significant and differential change not only in response to deletion of *ATX1* gene but also in response to changing copper levels without any significant interaction effect (G3). These genes were investigated in four different subgroups according to their responses to the perturbations.

The genes classified into G3.1 which, were down-regulated in response to the deletion of *ATX1* in comparison to the reference strain under both copper levels and up-regulated in response to high copper levels when compared to the copper deficient conditions in both strains, were found to be significantly (*p*-value < 5*10^−5^) enriched with cell-wall organization and endocytosis GO biological process terms. 17 genes (G3.2), which were down- and 22 genes (G3.3), which were up-regulated, respectively, both in response to the deletion of *ATX1* gene when compared to the reference strain and in response to high copper levels in comparison to copper deficient conditions were not associated with any biological process term. The manual inspection of these groups of genes revealed that the genes encoding proteins (Cyc7p, Hug1p) involved in mitochondrial electron transport and cell cycle arrest, respectively were down-regulated. The genes (*VHT1*, *PUT4 and MCH4)*, which encode transporters of H^+^-biotin, proline and monocarboxylic acid, respectively and the gene (*FMP23*) encoding a putative mitochondrial protein involved in iron-copper homeostasis were observed to be induced in response to *ATX1* deletion under both condition and in response to high copper condition in both strains. *VHT1* was previously reported to have higher mRNA levels in iron poor medium [20]. The 10 genes included into G3.4, were not identified to be significantly enriched with any process terms. 7 of these genes are not annotated with any GO Biological process term yet. YBL100W-A, and YBL005W-A, which are associated with transposition GO process term and BAT2, which encodes a cytosolic branched-chain amino acid aminotransferase were in this group.

### Interaction effect of changing copper levels and deletion of *ATX1* gene

The 2x2 factorial experimental design applied in this study enabled the identification of the genes, whose transcriptional responses to the deletion of *ATX1* gene were copper level dependent, or the genes, with diverse transcriptional responses to changing copper levels in the *ATX1* deleted and reference strains (G4).

**Fig. 2:**
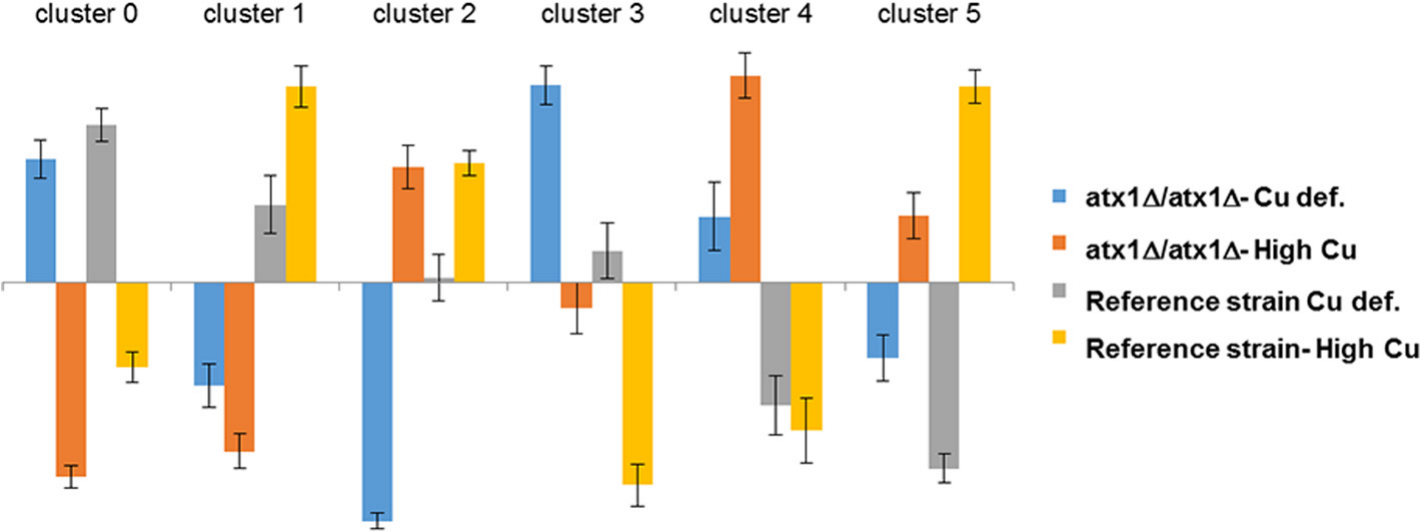
Clustering of the genes which show significant interaction effect. This figure contains the average normalized expression of the genes in each cluster that are significantly and differentially expressed in response to the interacting effect of gene deletion and copper level. Error bars indicate the confidence interval around the centroids. Blue and orange represents the average level of expression in the *ATX1* deleted cells under copper deficient and high copper containing conditions, respectively. Grey and yellow represents the average level of expression in the reference strain under copper deficient and high copper containing conditions, respectively

To further classify these transcripts according to their level of expression under studied conditions, Self-Organizing Maps (SOM) [18] was used for clustering. These transcripts could be clustered into 6 different clusters based on their level of expression and the enriched GO biological processes among these clusters were identified (Fig. 2).

The genes in cluster 0 were down-regulated under high levels of copper containing conditions when compared to copper deficient conditions in both strains; the repression in the *ATX1* deleted cells being more pronounced. These genes were significantly enriched with regulation of cell cycle and regulation of cyclin-dependent protein serine/threonine kinase activity process terms (*p*-value < 0.05). *PCL9*, which is a cyclin interacting with the Pho85 cyclin-dependent kinase that is expressed in a cell-cycle regulated manner in late M/early G1 phase of the cell cycle [21] was in this cluster. The lower expression levels of this gene under conditions containing high copper levels were also confirmed by real-time RT-qPCR analysis (Additional file 7).

The genes that showed lower expression in the *ATX1* deleted strain when compared to the reference strain under both copper levels were clustered in Cluster 1. These genes were also identified to be responding high copper levels when compared to copper deficient conditions in an opposite way in two strains. These genes were not identified to be significantly enriched with any process term but manual inspection of these genes revealed the presence of the genes associated with sterol transport (*NPC2*, *HES1*, *PRY3*) in this cluster. The gene encoding Sic1p, which is involved in G1 to S phase transition of mitotic cycle was also observed to be down-regulated in *ATX1* deleted yeast cells under both conditions. The analysis of the expression profile of *SIC1* via real-time RT-qPCR confirmed the down-regulation of this gene in the absence of *ATX1* under copper deficient conditions (FC = 1.84) (Additional file 7). Similarly *SCH9* encoding a substrate of Tor1p, which is involved in several biological processes including age dependent response to oxidative stress in chronological aging, regulation of transcription from RNA polymerase I, II and III, translation initiation and entry into G0 phase, was found to be repressed in *ATX1* deleted cells. The genes encoding a zinc responsive transcription factor (Zap1p) and a membrane protein (Izh4p) involved in the zinc ion homeostasis were also found within this cluster.

The genes, which were repressed in the *ATX1* deleted cells compared to the reference strain under copper deficient conditions but showed similar level of expression under high levels of copper containing conditions in these two strains, fall into Cluster 2. This cluster of genes was identified to be significantly enriched (*p*-value < 0.05) with cell differentiation, cell cycle and cytokinetic cell separation process terms. *ACE2*, which is a transcription factor that shows G2-to-M-phase-specific expression [22] was within this cluster. *ACE2* was also shown to play a role in regulating the basal-level expression of *CUP1* [23], encoding metallothionein that binds copper and mediates resistance to high concentrations of copper and cadmium. Further analysis of the expression level of this gene via real-time RT-qPCR showed that *ACE2* was respressed by 1.5 fold in the absence of *ATX1* gene in comparison to the reference strain not only under copper deficient conditions but also under high copper level conditions (Additional file 7). *ISU1* encoding a mitochondrial scaffold protein involved in cellular iron homeostasis and in the assembly of iron sulphur clusters and *BSD2* encoding a protein involved in heavy metal ion homeostasis were also among this group of genes.

The genes that were grouped in Cluster 3 were down-regulated under high levels of copper containing conditions in comparison to the copper deficient conditions in both strains. However, these genes showed the lower level of expression in the reference strain under high levels of copper containing conditions and higher level of expression in the *ATX1* deleted strain under copper deficient conditions. These genes were significantly enriched with mitotic sister chromatid cohesion and cation transport process terms (*p*-value < 0.05).

Cluster 4 consists of the genes that show higher expression in the *ATX1* deleted cells when compared to the reference strain under both conditions and also show higher induction in response to high copper levels when compared to copper deficient condition in the *ATX1* deletion strain. A detailed manual investigation of the this group indicated that several mitochondrial genes, including *PDH1* encoding a protein involved in respiration, *DIC1* encoding a dicarboxylic acid transporter, *YMC1* encoding a putative organic acid transporter, *PKP2* encoding a protein kinase, *EXO5* encoding a 5’-3’ exonuclease involved in mitochondrial genome maintenance, *MRM2* encoding a methyl transferase required for rRNA methylation and *GEM1* encoding a mitochondrial membrane GTPase are clustered into cluster 4. Gem1p is a subunit of ERMES complex that links ER to mitochondria and is involved in the inter-organelle calcium and phosphate exchange as well as mitochondrial DNA replication. The transcripts (SSA3, SPC1) associated with protein targeting to ER process term were also observed among the genes in cluster 4.

The genes which showed lower expression under copper deficient conditions in both strains and up-regulated under copper deficient conditions but down-regulated under high levels of copper containing conditions in the *ATX1* deleted cells when compared to the reference strain were clustered in Cluster 5. These genes were significantly enriched with indolalkylamine catabolic process, 'de novo' NAD biosynthetic process from tryptophan, cell wall biogenesis and oxidation reduction GO biological process terms.

### Integration of genetic interaction network with transcriptional response to *ATX1* deletion

Genetic interactions reflect the consequences of perturbing gene function and reveal relationships between diverse functional modules. Thus genetic interaction networks and its integration with other types of molecular data are reported to facilitate the identification of the rules governing the interdependence of genes in various cellular processes [24, 25]. In order to provide further insight into the functional organisation of the yeast cells in response to the deletion of *ATX1,* a genetic interaction network was constructed using the genes that were identified to be significantly expressed in response to the deletion of *ATX1* either dependent or independent of the copper levels (G1, G3 and G4). For the construction of the network, the genetic interactions in BioGrid database were used (Release 3.3.123) [15]. 430 of these genes were reported to have genetic interactions among each other yielding a connected network which contains 1223 interactions between 422 nodes (Additional file 8).

**Fig. 3:**
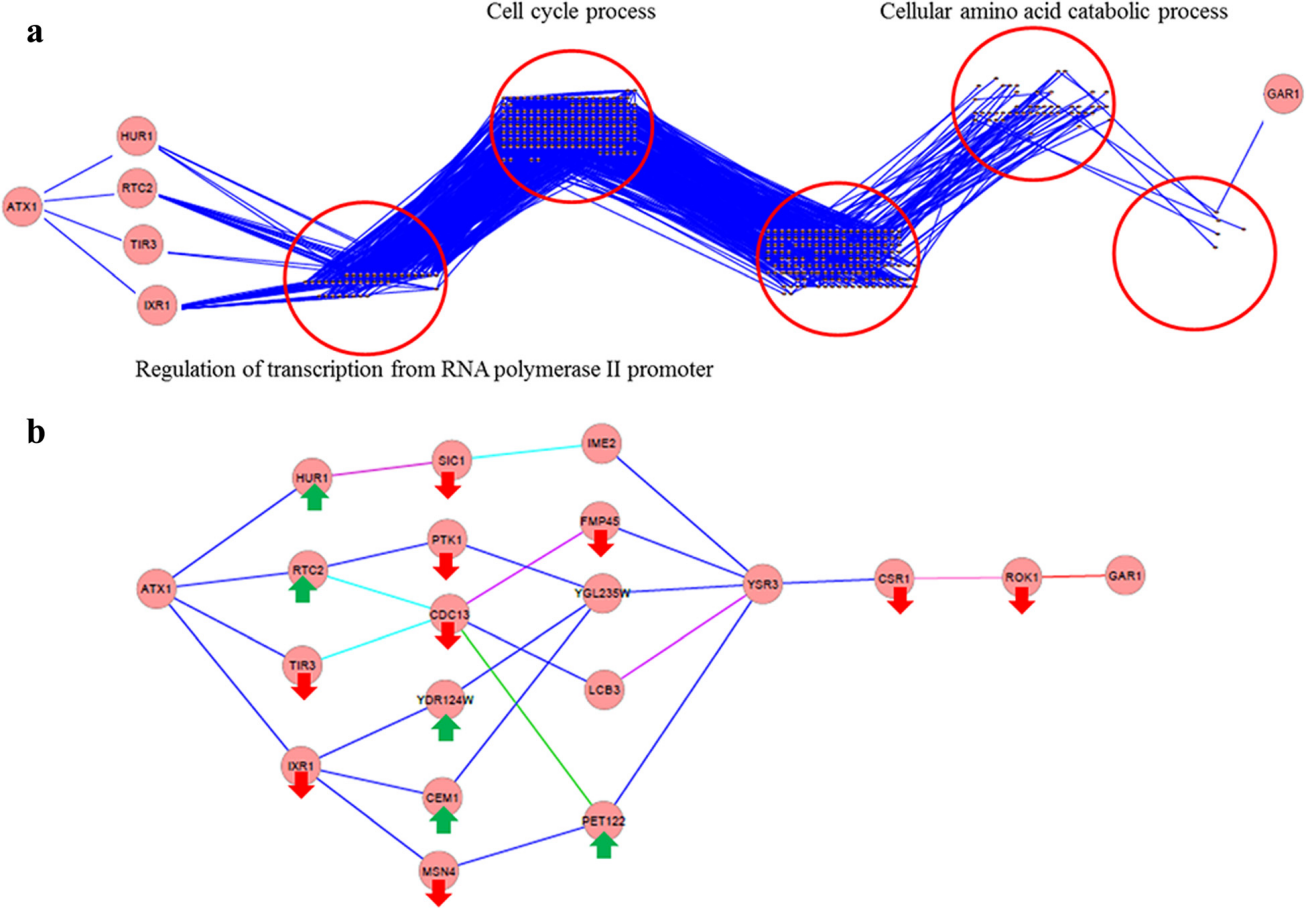
Integrative Analyses of Transcriptional Response to *ATX1* deletion with Genetic Interaction Network. This figure represents the connected component of the genetic interaction network between the genes responsive to the deletion of *ATX1* either dependent or independent of the copper level. **a** The genes were grouped according to their distance to *ATX1* and enriched GO biological process terms among each level of neighbours were indicated. **b** The paths connecting *ATX1* and *GAR1* are represented. The edges between the genes were coloured according to the type of the genetic interaction between the genes. Blue represents the negative genetic, purple represents the synthetic growth defect, green represents the positive genetic, red represents the synthetic lethal, pink represents the dosage lethality, and turquois represents the synthetic rescue. The genes that were either down- or up-regulated in the *ATX1* delatants were also indicated in the figure. The genes without any indication are the ones which gave opposite response to the gene deletion under different copper levels

In order to identify the relation between *ATX1* and the other genes, the nodes in the network were grouped according to their distance to *ATX1* (Fig. 3a)*.* This grouping revealed that the longest path between the *ATX1* and any gene in the network was 7. *ATX1* has direct genetic interactions with four genes; *IXR1*, which encodes HMG-domain protein which binds the major DNA adducts of the antitumor drug cisplatin [26], *TIR3*, which encodes a cell wall mannoprotein, *RTC2*, which encodes putative vacuolar membrane transporter for cationic amino acids, and *HUR1*, which encodes a protein with unknown function, null mutations of which reported to show decreased metal resistance and increased ionic stress resistance [27]. 36 genes, which were second neighbours of *ATX1*, were found to be significantly enriched with the regulation of transcription from RNA polymerase II promoter GO biological process terms. 161 genes, which were third neighbours of *ATX1*, were identified significantly enriched with cell cycle GO biological process term. There were 166 genes, which were the forth neighbour of *ATX1* but they were not significantly enriched with any GO biological process term. There were 48 genes, which were the fifth neighbours of the *ATX1* and they were significantly enriched with cellular amino acid catabolic GO biological process term. There were five genes, which were connected to *ATX1* as the 6th neighbours. *GAR1*, which encodes a protein involved in the modification and cleavage of the 18S pre-rRNA, was located at the longest distance to *ATX1*.

When the linear paths from *ATX1* to its longest distanced neighbour *GAR1* were identified (Fig. 3b), it was seen that either one of the first neighbours of the *ATX1* takes place in these linear paths, whereas only 6 and 5 of the second and third neighbours fall into these paths, respectively. *GAR1* was identified to be connected to third neighbour of *ATX1* through *ROK1*, *CSR1*, *YSR3*. Investigation of these paths revealed that the genes involved in lipid biosynthesis (*LCB3*, *CSR1*, *CEM1*, and *YSR3*), cell cycle (*SIC1*, *IME2*, *CDC13*, and *FMP45*) and rRNA processing (*ROK1*, *GAR1*) take place on the paths connecting *ATX1* to *GAR1*. Previously, lipid metabolism and cell cycle machinery were identified to be altered in the mouse model of Wilson disease [28]. Although, we previously didn’t identify a similar response in the *CCC2* deleted cells, an integrative analysis the transcriptional response to the deletion of *ATX1* highlighted the alteration in the expression of the genes involved in the cell cycle and lipid biosynthesis.

When the type of the genetic interactions was investigated, it was seen that they were dominated by negative genetic interactions indicating that the mutation or deletion of the two genes at the same time results in a more severe fitness defect or lethality. The response of these genes to the deletion of *ATX1* was also mapped on the graph whenever the response was not copper level dependent. However, a general trend between the type of the interaction and the direction of the change in the expression of the genes could not be observed.

A similar analysis was conducted for the complete genetic network for *S. cerevisiae*. This genetic interaction network was constructed for the genes, expression levels of which are studied in Yeast2 array. 5413 of those 5667 genes were reported to have genetic interactions among each other in BioGrid database (Release 3.3.123) [15], yielding a connected network with 5413 nodes and 173903 edges (Additional file 9). The nodes are grouped according to their distance to *ATX1* in 4 clusters. GO enrichment analysis among the genes in each cluster revealed that the second neighbours of *ATX1* were identified to be significantly enriched with cell cycle GO biological process terms. There were 2354 genes that have a connection to *ATX1* as a second neighbour and 417 of them were associated with cell cycle. This observation brought the question of whether enrichment of the cell cycle related genes in a network constructed only with the significantly and differentially expressed genes in response to *ATX1* deletion, is significant when this complete network was considered as the background, or not. To answer this question, the significance of the existence of the 44 cell cycle related genes among the 161 third neighbours of *ATX1* were calculated in a population of 2354 genes, 417 of which were associated with cell cycle using hypergeometric distribution function (*p*-value < 0.001). This analysis confirmed the significance of the previous finding and also provided additional evidence for the capacity of genetic interaction networks in underlying specific biological processes [24].

A similar integrative analyses were also conducted using the genes, which were reported to show a significant and differential expression in response to the deletion of *CCC2* gene [10]. However, since *CCC2* was not part of the connected network, a similar analysis could not be performed. When the nodes within the complete genetic network were grouped according to their distance to *CCC2*, the genes were clustered into four groups and none of these groups were enriched with cell cycle related processes. First neighbours of *CCC2* were identified to be significantly enriched with sphingolipid biosynthetic process (*p*-value < 0.01) and second neighbours were enriched with the terms related with vesicle-mediated transport. These findings also highlight the interdependence of *ATX1* and *CCC2* in different cellular processes.

### Integration of regulatory network with transcriptional response to *ATX1* deletion

An integrative approach was followed in order to elucidate the change in the landscape of the regulatory network in response to the deletion of *ATX1*. For this purpose, the transcription factors that are responsible from the transcriptional changes in response to deletion of *ATX1*, were identified. To do so, the transcription factors, that are reported to be the regulators of the genes, which are members of G1, G3 and G4, were identified. In this analysis, only the interactions with a documented evidence in Yeastract database were considered. 617 of the genes in G1, G3 and G4 were identified to have reported transcription factors with documented evidence in Yeastract database. Using a similar approach to GO-term enrichment analysis, enrichment of each TF among the regulators of these 617 genes was calculated using hypergeometric distribution function. All the available TF-gene interactions with a documented evidence for all the transcripts in transcriptome data was taken as the background (Additional file 10). Twenty transcription factors were identified to be significantly enriched among 290 transcription factors (Bonferroni corrected *p*-value < 0.0001). These transcription factors were Kar4p, Sok2p, Gcr2p, Yhp1p, Swi4p, Ste12p, Zap1p, Gcn4p, Tec1p, Bas1p, Hst1, Msi1, Msn2, Rap1, Sum1, Adr1, Phd1, Cup2, Fhl1, and Gzf3. Among these TFs, Msn2p, Zap1 and Gcn4p, are the regulators of the transcription from RNA polymerase II promoter in response to stress, zinc starvation and amino acid starvation respectively. Tec1p, Ste12p, Phd1p, and Sok2p are involved in the regulation of pseudohyphal growth. Kar4p regulates the genes in response to pheromones during meiotic nuclear division, Gzf3p is involved in catabolic gene expression and nitrogen catabolite repression, Rap1 is involved in chromatin silencing and in the activation of the glycolytic genes. Yhp1p, and Swi4p are transcription factors involved in mitotic cell cycle and Sum1p in negative regulation of transcription from RNA polymerase II promoter during mitosis. Fhl1p, Gcr2p are the regulators of ribosomal protein (RP) transcription. Cup2p is copper binding transcription factor and Adr1p is carbon-source responsive zinc-finger transcription factor involved in response to nutrient level. Hst1p and Msi1p are transcription factors involved in the negative regulation of mitotic recombination and DNA dependent nucleosome assembly respectively. This analysis revealed the predominance of the transcription factors involved in the stress response and regulation of cell growth among the enriched TFs.

## Discussion

### Atx1p; a potential role in the cell cycle regulation

Analysis of the global transcriptional response to the deletion of *ATX1* gene under changing copper levels in comparison to the reference strain indicated a possible role of *ATX1* gene in cell cycle related processes in *Saccharomyces cerevisiae*. Identification of the enriched process terms among the significantly and differentially expressed genes together with the detailed manual inspection of these genes showed that the transcripts related with cell cycle and it’s transcriptional regulation were affected from the deletion of *ATX1* gene either irrespective of the copper level or conditionally. Absence of *ATX1* resulted in the repression of the genes involved in the regulation of cell cycle and induction of the genes associated with meiotic cell cycle irrespective of the copper level. The genes that play a role in the cell cycle, cell differentiation and cytokinetic cell separation processes were repressed in the *ATX1* deleted cells under copper deficient conditions but not under high levels of copper containing conditions. In mammalian cells, *ATOX1*, which is the ortholog of *ATX1*, was reported to play a role in copper stimulated cyclin D1 expression and cell proliferation [29]. *ATOX1* also identified to play an important role in the copper-stimulated proliferation of non-small lung cancer cells and to be a potential therapeutic target for lung cancer therapy targeting copper metabolism [30]. Although *ATX1* gene has not been previously associated with cell cycle in yeast, the alteration in the transcriptional response of the cell cycle associated genes to different copper levels in the absence of *ATX1* gene might indicate a possible role of this protein in cell proliferation. Moreover, identification of *CLN3*, which is the G1 cyclin involved in cell cycle progression in yeast, to be down-regulated in the *ATX1* deletion mutant in comparison to the reference strain under high levels of copper containing also provided additional support for this hypothesis. The down-regulation of the genes encoding Ime1p which is the master regulator of meiosis, Cdc39p which is involved in cell cycle regulation and Msa1p and Msa2p which are activators of G1 specific transcription factors in response to *ATX1* deletion independent of the conditions were also supporting that Atx1p has an additional function in the yeast cells. Furthermore, integrative analysis of genetic interaction network and transcriptome revealed that *IXR1*, which encodes the protein binding the major DNA adducts of the antitumor drug cisplatin that is used in adjuvant chemotherapy for non-small-cell lung cancer [31], was among the first neighbours of *ATX1* and down-regulated in the *ATX1* deleted cells irrespective of the copper level in comparison to the reference strain.

Identification of the genetic interactions between the genes that are significantly expressed in response to the deletion of *ATX1* also revealed that genes involved in cell cycle related process were affected from the absence of *ATX1*. Analysis of the network components according to their distance to *ATX1* showed the genes involved in cell cycle do not have any reported direct genetic interaction with *ATX1*. However, the interaction between these cell cycle related genes and the ones that are involved in the regulation of transcription that are second neighbours of *ATX1* might indicate the possible regulatory role of *ATX1* in the cell cycle related processes. Furthermore, integration of the regulatory network with transcriptome also highlighted that the existence of the TFs involved in the regulation of cell growth and cell cycle related genes, among the enriched transcription factors.

The genes associated with the cell wall biogenesis and endocytosis process terms were identified to be regulated at transcriptional level both by changing copper levels and also by the deletion of the *ATX1* gene. The genes involved in cell wall biogenesis were reported to be regulated in response to cell wall stress and also through cell cycle [32]. The genes associated with cell wall biogenesis process term were identified to be up-regulated in response to high copper levels in comparison to copper deficient conditions in both strains and also down-regulated in the *ATX1* deleted cells when compared to the reference strain under both copper levels. The deletion of *ATX1* gene and changing copper levels were identified to have an interactive effect on the transcription of another group of genes associated with cell wall biogenesis. These genes were down-regulated under high levels of copper containing conditions but up-regulated under copper deficient conditions in the *ATX1* deleted strain compared to the reference strain. Up-regulation of the genes involved in cell wall biogenesis in response to high copper levels can be interpreted as a response to oxidative stress [33] to mediate resistance to high copper concentrations [34]. The copper level dependent response observed in the *ATX1* deleted cells may also be explained by the cell cycle dependent regulation of the genes involved in cell wall biogenesis.

It was previously shown that G_2_/M-phase cells were more copper sensitive than G_1_/S-phase cells and *SOD1* was required for cell-cycle dependent Cu resistance [35]. Overexpression of the *ATX1* was previously reported to substitute for *SOD1* by supressing the oxidative damage [36]. Recently, the molecular link between excess copper levels and the control of cell cycle and, its relation with the changes in redox balance and ROS accumulation that regulate cell proliferation was also reported in mammary epithelial cells [37]. It was shown that cells response to copper treatment by activating Cyclin D1 and B1 and by increasing the levels of reduced intracellular glutathione (GSH), decreasing reactive oxygen species (ROS) generation. Copper induced Cyclin D1 expression was shown to be completely abolished in mouse embryonic fibroblasts lacking *ATOX1* [29]. The analysis of the cell-cycle dependent Cu resistance in the *SOD1* deleted yeast cell when *ATX1* was overexpressed might give a more comprehensive picture about the link between copper resistance and cell cycle and the role of *ATX1* in these processes.

### Atx1p and Ccc2p; two proteins with consecutive roles in copper transport but different output in their absence

Atx1p is known as a cytosolic copper metallochaperone required for copper-dependent iron absorption although a second endocytosis-dependent pathway other than Atx1p that delivers copper to Ccc2p was previously reported [4]. Yeast cells showed either decreased or complete respiratory deficiency in the absence of *ATX1* and *CCC2* genes, respectively. This respiratory defect was shown to be recovered in the presence of high copper levels. Therefore, the transcriptional re-organisation in response to the absence of these genes was studied under copper deficient and high copper conditions to better understand the roles of these two proteins.

Previously, iron metabolism was reported to be impaired in the absence of *CCC2* gene under copper deficient conditions leading to iron deficiency under this condition. The transcriptional response observed in the iron transport genes in response to changing copper levels were identified to be antagonistic in the reference strain and *CCC2* deleted cells [10]. On the other hand, the genes involved in the iron ion homeostasis showed a similar response to changing copper levels in the reference and *ATX1* deleted cell. A decreased iron absorption in the *ATX1* deleted cells was previously reported [4] but under studied conditions no significant difference was identified in the intracellular and extracellular iron levels between the *ATX1* deleted cells and the reference strain. Furthermore, the genes involved in iron transport were not identified to be responsive to the deletion of *ATX1* at transcriptional level. This might be explained by the incomplete blockage of iron uptake in the absence of *ATX1* gene, whereas complete inhibition of transfer in the case of *CCC2* deletion [4]. This finding also supported the fact that an Atx1p independent pathway transfers copper to Ccc2p for further incorporation to iron transporter; Fet3p.

Endocytosis was previously proposed as a possible *ATX1* independent pathway that transfers copper to Ccc2p. The analysis of the genes that are determined to be differentially and significantly expressed in this study revealed that endocytosis GO biological process term was enriched among different groups. In order to elucidate the overall change in this process, the genes that are associated with this term and also identified to be significantly and differentially expressed in this study were identified. There were 33 genes that were associated with endocytosis and showed a significant and differential change at transcriptional level. 9 of them (*INP52, ART5, COS10, ARV1, PAN1, HRR25, INP53, MYO5, YPT35*) were responsive to the deletion of *ATX1* only. 10 of those (*YPT53, ROG3, FTH1, MON2, ENT1, PRK1, YPK1, RIM8, AKL1, ATG20*) were responsive to copper level only. There were 10 (*LAS17, SCD5, ENT2, SNX41, CSR2, MYO3, SDS24, VRP1, ARK1, ROD1*) and 4 (*HES1, JJJ1, SVL3, SUR7*) genes that were identified to be significantly and differentially expressed in response to additive and interaction effect of the perturbations, respectively. However, none of these genes were reported to have a physical interaction either with Ctr1p or Ccc2p.

On the other hand, up-regulation of *MTM1*, which encodes a mitochondrial protein that is involved in mitochondrial iron homeostasis together with the up-regulation of several genes encoding mitochondrial proteins in response to deletion of *ATX1*, might indicate a possible disturbance in the mitochondrial iron homeostasis.

The genes involved in the NAD+ biosynthesis from tryptophan were previously identified to be affected from the absence of *CCC2* gene and also from the change in copper levels. The induction of *BNA2* and *BNA4 u*nder copper deficient conditions in the *CCC2* deleted cells was previously interpreted as a transcriptional re-organisation of the respiratory deficient *CCC2* deleted cells to maintain NAD+/NADH ratio. Previously, we have also reported that *BNA2* and *BNA4* were down-regulated under high levels of copper containing conditions in *CCC2* deleted strain in contrast to the response observed in the reference strain. In the *ATX1* deleted cells, expression levels of *BNA2*, *BNA4* and *BNA1* were identified to be induced in response to high copper levels as it was the case in the reference strain. Under copper deficient conditions, the expression level of *BNA2* gene was repressed more than 1.5 fold in *ATX1* deleted cells when compared to the reference strain and *BNA1* and *BNA4* were expressed almost at similar levels in these two strains. The difference in this transcriptional response might be explained by the much milder decreased respiratory capacity in the *ATX1* deleted cells in comparison to the *CCC2* deleted cells.

## Conclusion

In this study, we have investigated the effect of deletion *ATX1* at transcriptional level under two different copper levels. This study revealed the differences between the transcriptional reorganisation in response to the absence of Cu (+2)-transporting P-type ATPase; Ccc2p and cytosolic copper metallochaperone; Atx1p, although these proteins play consecutive functions in the intracellular transport of copper. This result provided additional evidence for the existence of an *ATX1* independent pathway that transfers copper to Ccc2p. Furthermore, the analysis of transcriptional response to the deletion of *ATX1* and also the integrative analysis of the transcriptome with genetic interaction and regulatory network revealed that the genes involved in the cell cycle regulation were significantly affected from the absence of *ATX1.* This finding indicated for the first time, a possible regulatory role of *ATX1* in cell cycle regulation, likewise its mammalian counterpart *ATOX1*. We believe that the observation of involvement of *ATX1* in cell cycle regulation may shed light on the potential of *Saccharomyces cerevisiae* as a model organism to study the capacity of *ATOX1* as a therapeutic target for lung cancer therapy. Further investigations including analysis of transcriptomic changes in response to over-expression of *ATX1* under different copper conditions will be required to enlighten the molecular mechanism of the regulatory role of Atx1p in the regulation of cell cycle. These studies will also possibly elucidate the down-regulation of the genes encoding transcriptional factors as well as that of the genes encoding proteins involved in RNA 3’-end processing, in response to the *ATX1* gene deletion independent of extracellular conditions.

## Additional files

Additional file 1: The sequences of the reverse and forward primers of the genes of interest and the housekeeping genes. (XLSX 10 kb) Additional file 2: The raw Cq values obtained for each gene for each sample. The values were provided as the average of the duplicate assays. (XLSX 9 kb) Additional file 3: Spot assay to determine the respiratory capacity of the deletion mutants in comparison to reference strain. This figure represents the spot assay conducted using the reference strain, *ATX1* deleted cells and *CCC2* deleted cells under two different conditions; YPG, as control and 0.5 mM copper containing YPG, to show the effect of copper supplementation on the respiratory capacity of the cells. (JPG 36 kb) Additional file 4: The genes that were identified as significantly and differentially expressed in this study. The lists of the genes that have been identified to be significantly and differentially expressed in response to mutant effect (G1), copper effect (G2), additive effect (G3) and interaction effect (G4) are provided in the corresponding sheets together with the enriched GO process terms within in each group. (XLSX 446 kb) Additional file 5: Differential expression of genes that are responsive to *ATX1* deletion. The values represent the fold changes between the reference strain and the *ATX1* deleted strain under corresponding conditions. Fold changes were calculated by dividing the average expression level obtained for the reference strain under corresponding condition by that of obtained for the *ATX1* deletion mutant via either real-time RT-qPCR or microarray analysis. The values higher than 1.5 represent the differential down-regulation in the absence of *ATX1* gene. (DOCX 12 kb) Additional file 6: Intracellular and extracellular iron levels. This figure represents the intracellular (blue) and extracellular (red) iron levels in the reference and *ATX1* deleted cells under copper deficient and high copper conditions. Error bars show the standard deviation. (TIF 59 kb) Additional file 7: Differential expression of genes that are differentially expressed in response to interaction effect of gene deletion and copper level. The fold change values obtained between high copper and copper deficient conditions in the reference strain or *ATX1* deleted cells and between *ATX1* deleted cells and the reference strain under copper deficient or high copper conditions for a) *PCL9* b) *SIC1* c) *ACE2*. Blue bars represent the fold changes calculated using the expression profiles obtained via real-time RT-qPCR and red bars represent the fold changes calculated using the expression levels obtained via microarray analysis. (TIF 189 kb) Additional file 8: Genetic interaction within significant set. The genetic interaction network constructed using the genetic interactions within the genes in G1, G3 and G4 are provided in this file. The list of the nodes grouped according to their distance to *ATX1* is also provided. (XLSX 778 kb) Additional file 9: The complete genetic interaction network. The list of the nodes grouped according to their distance to *ATX1* and *CCC2* is provided in this file. (XLSX 6197 kb) Additional file 10: Enriched TFs. The regulatory network and the result of the TF enrichment analysis are provided in the file. (XLSX 2909 kb)
