# Supplementary figures and images for: Transcriptomic response of yeast cells to ATX1 deletion under different copper levels
Source: BMC Genomics. 2016 Jul 11;17:489. doi: 10.1186/s12864-016-2771-6 (PMC4940881; doi:10.1186/s12864-016-2771-6)

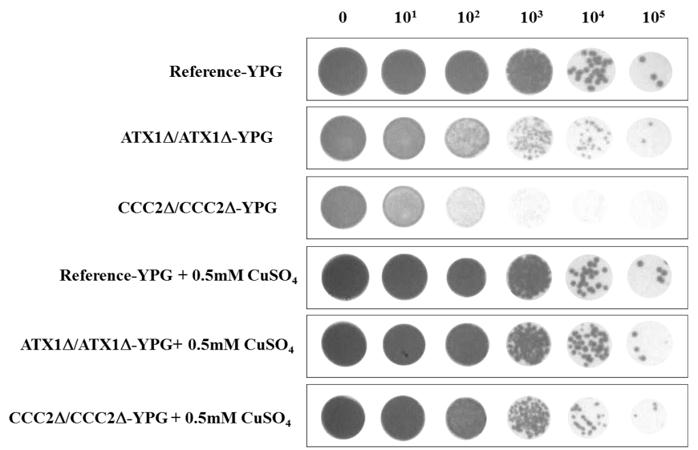

Supplement: Additional file 3: — Spot assay to determine the respiratory capacity of the deletion mutants in comparison to reference strain. This figure represents the spot assay conducted using the reference strain, ATX1 deleted cells and CCC2 deleted cells under two different conditions; YPG, as control and 0.5 mM copper containing YPG, to show the effect of copper supplementation on the respiratory capacity of the cells. (JPG 36 kb) [file 12864_2016_2771_MOESM3_ESM.jpg]

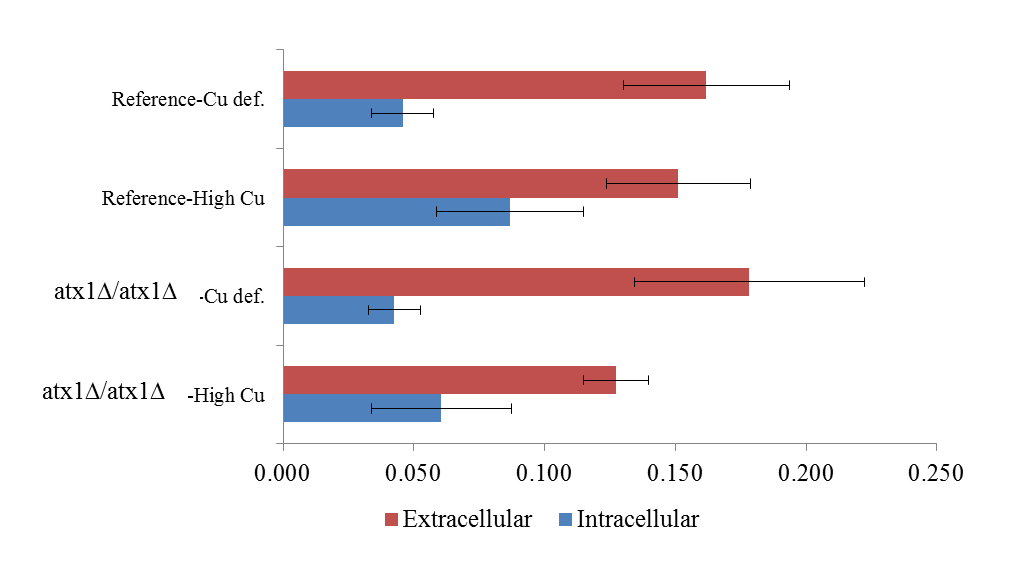

Supplement: Additional file 6: — Intracellular and extracellular iron levels. This figure represents the intracellular (blue) and extracellular (red) iron levels in the reference and ATX1 deleted cells under copper deficient and high copper conditions. Error bars show the standard deviation. (TIF 59 kb) [file 12864_2016_2771_MOESM6_ESM.tif]

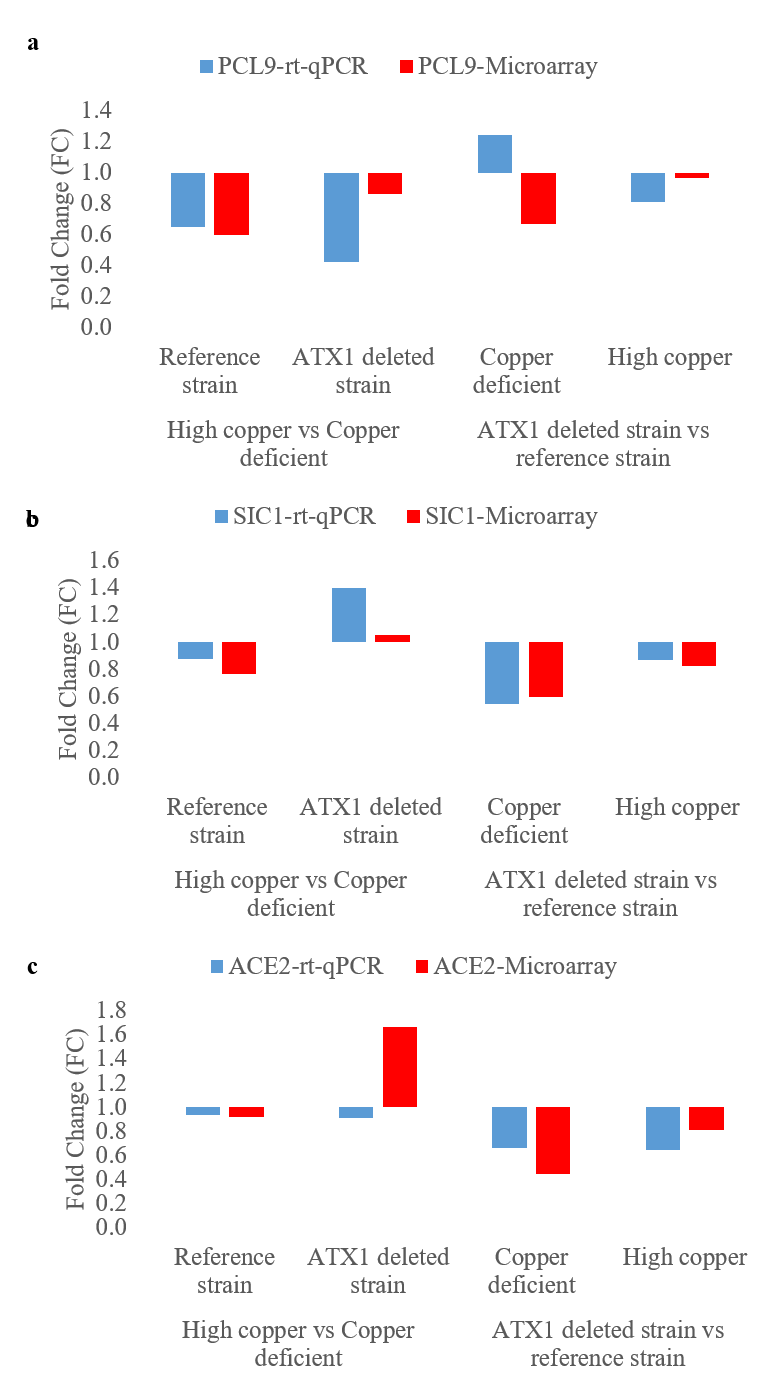

Supplement: Additional file 7: — Differential expression of genes that are differentially expressed in response to interaction effect of gene deletion and copper level. The fold change values obtained between high copper and copper deficient conditions in the reference strain or ATX1 deleted cells and between ATX1 deleted cells and the reference strain under copper deficient or high copper conditions for a) PCL9 b) SIC1 c) ACE2. Blue bars represent the fold changes calculated using the expression profiles obtained via real-time RT-qPCR and red bars represent the fold changes calculated using the expression levels obtained via microarray analysis. (TIF 189 kb) [file 12864_2016_2771_MOESM7_ESM.tif]
