# Supplementary material for: Transcriptomic response of yeast cells to ATX1 deletion under different copper levels
Source: BMC Genomics. 2016 Jul 11;17:489. doi: 10.1186/s12864-016-2771-6 (PMC4940881; doi:10.1186/s12864-016-2771-6)
Supplement: Additional file 5: — Differential expression of genes that are responsive to ATX1 deletion. The values represent the fold changes between the reference strain and the ATX1 deleted strain under corresponding conditions. Fold changes were calculated by dividing the average expression level obtained for the reference strain under corresponding condition by that of obtained for the ATX1 deletion mutant via either real-time RT-qPCR or microarray analysis. The values higher than 1.5 represent the differential down-regulation in the absence of ATX1 gene. (DOCX 12 kb) [file 12864_2016_2771_MOESM5_ESM.docx]

Additional File 5. RT-qPCR confirmation of genes that are responsive to the deletion of *ATX1* gene

|  | rt-qPCR | | Microarray | |
| --- | --- | --- | --- | --- |
|  | Copper deficient | High copper | Copper deficient | High Copper |
| CLN3 | 0.99 | 5.88 | 1.89 | 1.95 |
| IME1 | 1.70 | 1.69 | 1.73 | 1.99 |
| MSA1 | 2.24 | 1.21 | 1.63 | 1.26 |
| MSA2 | 3.46 | 53.07 | 1.55 | 1.50 |
